# Cyclic azapeptide CD36 ligand attenuates cardiac injury and reduces long‐chain fatty acid accumulation after myocardial ischemia–reperfusion in mice

**DOI:** 10.1002/2211-5463.70265

**Published:** 2026-05-07

**Authors:** Jade Gauvin, Naghme Radmannia, David N. Huynh, Liliane Ménard, Caroline Daneault, Maïté Veilleux, Ahsanullah Ahsanullah, André C. Carpentier, William D. Lubell, Matthieu Ruiz, Huy Ong, Sylvie Marleau, Simon‐Pierre Gravel

**Affiliations:** ^1^ Faculty of Pharmacy Université de Montréal Canada; ^2^ Metabolomics Platform, Research Center, Montreal Heart Institute Université de Montréal Canada; ^3^ Department of Chemistry Université de Montréal Canada; ^4^ Division of Endocrinology, Department of Medicine Université de Sherbrooke, Centre de Recherche du CHUS Canada; ^5^ Faculty of Medicine, Department of Nutrition Université de Montréal Canada; ^6^ Institute for Research in Immunology and Cancer Université de Montréal Canada

**Keywords:** azapeptide, CD36, lipidomics, long‐chain fatty acids, metabolomics, myocardial ischemia and reperfusion

## Abstract

Ischemic heart disease remains a leading global cause of death. We investigated the cardioprotective effects of the selective cluster of differentiation‐36 receptor (CD36) modulator azapeptide MPE‐298 in a mouse model of myocardial ischemia–reperfusion. Given before reperfusion, a single intravenous dose of azapeptide MPE‐298 reduced infarct size by 44% of the area‐at‐risk and transiently decreased left ventricular long‐chain fatty acids (LCFA) accumulation, independently of saturation status. Metabolomic profiling identified changes in amino acids that may fuel the tricarboxylic acid cycle and provide substrates for glutathione‐dependent antioxidant defense. Gene expression analysis showed transient modulation of oxidative stress and inflammation‐associated genes in both heart and adipose tissue. Thus, we conclude that modulation of CD36 by azapeptide MPE‐298 exhibits therapeutic potential for treating acute myocardial ischemia and reperfusion by supporting metabolic recovery and limiting excess LCFA uptake.

## Graphical abstract

In a murine model of myocardial ischemia and reperfusion (MI/R), the CD36 azapeptide ligand MPE‐298 reduces cardiac injury and transiently lowers left ventricular long‐chain fatty acids (LCFAs) accumulation 3 h after reperfusion, accompanied by a decrease of oxidative stress and inflammation‐associated genes' expression in the heart and adipose tissue.

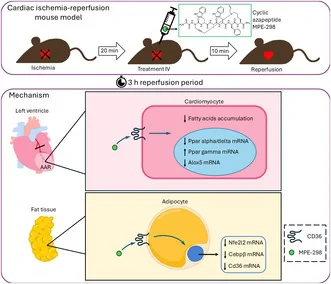

**Abbreviations**
5‐LO: 5‐lipoxygenase
AAR: area at risk
Alox12: arachidonate 12‐lipoxygenase
Alox15: arachidonate 15‐lipoxygenase
Alox5: arachidonate 5‐lipoxygenase
CCL2: CC motif chemokine ligand 2
CD36: cluster of differentiation 36
Cebpβ: CCAAT/enhancer‐binding protein beta
Ct: cycle threshold
DEPC: diethylpyrocarbonate
DHA: docosahexaenoic acid
dNTP: deoxynucleoside triphosphate
DPA: docosapentaenoic
ETE: eicosatrienoic acid
FA: fatty acids
FAMES: fatty acid methyl esters
GHRPs: growth hormone‐releasing peptides
IA: infarct area
IHD: ischemic heart disease
IV: intravenous or intravenously
Ki: inhibition constant
LCAL: left coronary artery ligation
LCFA: long‐chain fatty acids
LOX‐1: lectin‐like oxidized low‐density lipoprotein receptor‐1
LTB_4_
: leukotriene B_4_
LV: left ventricle or left ventricular
MI: myocardial infarction
MI/R: myocardial ischemia and reperfusion
MMLV: Moloney murine leukemia virus
MUFA: monounsaturated fatty acids
NEFA: nonesterified fatty acids
Nfe2l2: nuclear factor erythroid‐derived 2‐like 2
NOX: NADPH oxidase
Nrf2: nuclear factor erythroid‐2 related factor 2
oxLDL: oxidized low‐density lipoproteins
PCI: positive chemical ionization
PPAR: peroxisome proliferator‐activated receptor
PUFA: polyunsaturated fatty acids
qPCR: quantitative polymerase chain reaction
R‐FSL‐1: R‐fibroblast‐stimulating lipopeptide‐1
ROS: reactive oxygen species
SC: subcutaneous
SFA: saturated fatty acids
TCA: tricarboxylic acid
TNF‐α: tumor necrosis factor‐α
TTC: 2,3,5‐triphenyltetrazolium chloride
μPET: micro‐positron emission tomography

The incidence of cardiovascular diseases, particularly ischemic heart disease (IHD) and stroke, continues to rise globally despite progress in prevention, treatment, and monitoring [1]. Deaths related to IHD are expected to persist, largely due to aging populations and the increasing prevalence of hypertension, diabetes, and obesity [2, 3, 4] despite reported declines in certain high‐income countries [5]. The alarming rise in younger and middle‐aged individuals suffering IHD and myocardial infarction (MI) is associated with significant morbidity, psychological effects, and financial strain on patients and families [6]. Among those surviving MI, up to 30% will develop heart failure, despite implementation of pharmacological interventions [7]. Novel therapeutic approaches to treat cardiovascular diseases are urgently needed to improve the limited long‐term effectiveness of current strategies, particularly in the context of prevalent comorbidities and an aging population.

Timely clinical reperfusion is crucial for preserving myocardial tissue and preventing irreversible damage following ischemia [8, 9]. Reperfusion, however, may aggravate tissue injury, due in part to pronounced oxidative and inflammatory consequences [8]. Metabolic disturbances following reperfusion, particularly elevated levels of circulating nonesterified fatty acids (NEFA), can impair recovery of cardiac function [10]. Rapid mobilization of NEFA from adipose tissue following myocardial ischemia and reperfusion (MI/R) induces a detrimental metabolic shift by elevating myocardial long‐chain fatty acids (LCFA) availability during early reperfusion, a process partly regulated by the cluster of differentiation 36 (CD36) receptor [11]. In this vulnerable state, myocardial β‐oxidation produces ATP less efficiently than glucose metabolism and disrupts the coupling between glycolysis and pyruvate oxidation, resulting in cardiomyocyte acidification, ionic imbalances, reduced cardiac efficiency, and impaired functional recovery [10].

CD36 is a class B2 scavenger receptor expressed by a broad range of immune and non‐immune cells, in which it mediates different cell type‐specific functions [12]. It plays a central role in immunometabolism by integrating metabolic and immune signaling in response to a variety of extracellular ligands, including thrombospondin domain–containing proteins, danger‐ and pathogen‐associated molecular patterns, LCFA, and oxidized low‐density lipoproteins (oxLDL) [12]. In the heart, CD36 is essential for the uptake of free LCFA across cardiomyocyte plasma membranes, thereby coupling fatty acids (FA) transport with myocardial metabolic activity [13, 14]. Growth hormone (GH)‐releasing peptides (GHRPs) were initially shown to bind the CD36 protein in murine cardiac membranes [15], which led to exploration of non‐GH‐secreting GHRP analogs for possible cardiovascular effects. In an atherosclerotic apolipoprotein E‐deficient mouse model fed a high‐fat high‐cholesterol diet, GHRP‐6 analogs and azapeptide derivatives reduced the progression of atherosclerosis [16] and promoted the regression of aortic lesions [17, 18]. These peptides acted mainly on intimal macrophages, reducing oxLDL uptake, enhancing cholesterol efflux and reverse transport, and lowering vascular inflammation in a CD36‐dependent manner [16, 17, 18, 19, 20, 21]. In the heart, our previous studies showed that a 14‐day pretreatment with a CD36 modulator favorably altered myocardial metabolism and improved dysfunction after transient myocardial ischemia in mice [11].

However, the use of GHRPs such as hexarelin (H‐His‐D‐2‐Methyl‐Trp‐Ala‐Trp‐D‐Phe‐Lys‐NH_2_) as CD36 ligands was limited in further studies due to their unselective binding to the ghrelin receptor [22]. In contrast, azapeptide derivatives of GHRP‐6 have demonstrated high and selective binding affinity for CD36, as evidenced by an inhibition constant (Ki) of 100 nm, determined in a competition assay [23, 24]. Moreover, cyclic azapeptides, such as MPE‐298, demonstrated marked potency in suppressing pro‐inflammatory nitric oxide and cytokine production, including tumor necrosis factor‐α (TNF‐α) and the CC motif chemokine ligand 2 (CCL2), in macrophages stimulated with a Toll‐like receptor‐2 agonist, R‐fibroblast‐stimulating lipopeptide‐1 (R‐FSL‐1), achieving these effects at lower concentrations (10^−7^ m) than those required for their linear counterparts [25]. In macrophages, azapeptide MPE‐298 was shown to bind and induce CD36 endocytosis through activation of the Lyn (Src family) and Syk (spleen) tyrosine kinases [26]. The internalized CD36‐MPE‐298 complex then inhibits lectin‐like oxLDL receptor‐1 (LOX‐1)‐mediated signaling triggered by oxLDL, thereby reducing CCL2 secretion and preventing both mitochondrial membrane potential depolarization and reactive oxygen species (ROS) production.

Prior work with CD36 ligands primarily relied on pretreatment protocols, and the direct impact of an acute intervention shortly before reperfusion remained undetermined. In this study, we examined whether a single intravenous dose of MPE‐298, administered before reperfusion during left coronary artery ligation (LCAL) in C57BL/6 mice, could reduce myocardial injury, alter myocardial energetics and metabolic substrate accumulation in the left ventricle (LV) as monitored using lipidomic and metabolomic profiling, as well as modulate CD36‐related signaling pathways.

## Materials and methods

### Chemicals

Azapeptide MPE‐298 was prepared by solid‐phase synthesis employing an A^3^‐macrocyclization as detailed previously [27]. Binding affinity was assessed using a competitive binding assay, in which MPE‐298 was identified as the azapeptide with the highest affinity (0.08 μm) among other synthetized analogs of GHRP‐6 [25, 27]. Potency was evaluated by measuring inhibition of the R‐FSL‐1‐stimulated inflammatory response in RAW264.7 murine macrophages, where MPE‐298 reduced the secretion of TNF‐α and CCL2, as determined using commercial ELISA kits (mouse ELISA Ready‐SET‐Go!™ kits; eBioscience, San Diego, CA, USA).

### Animals

The wild‐type C57BL/6J inbred mouse strain (stock number 000664) (*Cd36* ^+/+^) and CD36‐deficient (*Cd36* ^−/−^) mice (stock number 019006) were purchased from the Jackson Laboratory (Bar Harbor, ME, US) and bred in a specific pathogen‐free (SPF) facility. Mice were housed in ventilated cages before being transferred to a controlled environment in static cages. Mice were maintained on a 12 : 12 h light/dark cycle and fed Teklad irradiated global chow (T.2918.15; Inotiv Inc., West Lafayette, IN, US) and water *ad libitum*. All experimental protocols received approval from the Institutional Animal Ethics Committee (No. 23‐034) and were conducted in accordance with the guidelines set forth by the Canadian Council on Animal Care and the US National Institutes of Health.

### Experimental groups and protocols

Aged‐matched male *Cd36* ^+/+^ and *Cd36* ^−/−^ mice (30–35 g) were randomly assigned to one of two main experimental protocols: mice underwent either a 30 min LCAL surgery or a corresponding sham operation. Myocardial infarct area (IA) was evaluated 24 h after reperfusion in both *Cd36* ^+/+^and *Cd36* ^−/−^ mice. In a separate cohort, *Cd36* ^+/+^ mice were subjected to LCAL and reperfused for either 3 or 24 h to enable metabolomic and lipidomic analysis of the LV.

LCAL was performed following the protocol detailed previously [11] with some modifications. Mice were initially anesthetized with 3.5% isoflurane in 1.5 L·min^−1^ O_2_, intubated and given 6 mg·kg^−1^ intraperitoneal lidocaine to prevent arrhythmias. Electrocardiography was monitored, and body temperature maintained with a heating pad. Subcutaneous (SC) administration of a 0.9% NaCl–2.5% dextrose solution (10 mL·kg^−1^) was given for the first hour, then 5 mL·kg^−1^ as needed. Ophthalmic ointment was applied to prevent corneal dryness. Surgery was performed under a Nikon SMZ645 stereomicroscope (0.8–5X magnification). A left thoracotomy was performed at the third intercostal space. The transverse and deep pectoral muscles were separated using a retractor to expose the heart. The left coronary artery was visualized under a light block and ligated 1 mm distal to the left atrial appendage using a 1/2 circle spatula needle and an 8–0 nylon thread was tied over a small piece of Silastic tubing. After ligation, ischemia was maintained for 30 min during which the at‐risk region became visibly pale. Ten minutes before reperfusion, mice received an IV injection of 150 μL of vehicle (0.9% NaCl) or MPE‐298 (3 μmol·kg^−1^). After ligation release, the surgical site was sutured, and long‐acting buprenorphine (1 mg·kg^−1^) was administered subcutaneously followed by extubation. At 3 or 24 h after reperfusion, mice were euthanized by an overdose of isoflurane anesthesia followed by exsanguination. Sham‐operated mice underwent the same procedure without ligature placement.

### Determination of the myocardial area at risk and infarct size

Twenty‐four hours after reperfusion, mice were anesthetized and re‐ligated at the original site of LCAL. A 0.5 mL IV injection of 2.0% Evans blue dye (Sigma‐Aldrich, St‐Louis, MO, US) was injected retrogradely through the aorta to delineate nonischemic (blue‐stained) tissue. Hearts were excised, rinsed with PBS, snap‐frozen, and the LV sectioned transversely into 1 mm sections using a mouse heart slicer matrix. Slices were incubated with 2,3,5‐triphenyltetrazolium chloride (TTC) at a concentration of 1% at 37 °C for 15 min to visualize IA and then fixed in 10% neutral buffered formalin for 12 h. Each slice was weighed and imaged on both sides using a stereomicroscope‐mounted digital camera (Coolpix 4500; Nikon). The LV area, area at risk (AAR), and IA were quantified using computerized planimetry with Adobe Photoshop CC 2015, and analysis of treated and untreated groups was performed blindly. Results from each slice were averaged using the area‐weighted mean approach, calculated as (A_1_ × W_1_) + (A_2_ × W_2_) + … + (A_n_ × W_n_), in which A is the IA percentage determined by planimetry, and W is the weight of the corresponding slice [11].

### Targeted fatty acid methyl esters analysis

The LV was processed for quantitative profiling of bounded FA using gas chromatography–mass spectrometry, following established methods [28, 29]. Briefly, approximately 50 mg of pulverized tissue was incubated overnight at 4 °C in a 2 : 1 chloroform/methanol solution containing 0.004% butylated hydroxytoluene (BHT), filtered and dried under nitrogen gas. FA were analyzed as methyl esters (FAMES) which were prepared by transesterification using acetyl chloride in methanol. The analysis was performed on a 7890B gas chromatograph coupled to a 5977A Mass Selective Detector (Agilent Technologies, Santa Clara, USA), using a J&W Select FAME CP7420 capillary column (100 m × 250 μm inner diameter; Agilent Technologies Inc.) and operated in positive chemical ionization (PCI) mode with ammonia as the reagent gas. Samples were processed under the following conditions: injection at 270 °C in split mode (split ratio: 50 : 1) and using high‐purity helium as the carrier gas at a constant flow rate of 0.44 mL·min^−1^. The temperature gradient was set to 190 °C for 25 min, followed by an increase of 1.5 °C·min^−1^ until reaching 236 °C. FA were analyzed as [M + NH_3_]^+^ ions, and concentrations were determined using standard curves and isotope‐labeled internal standards.

### Targeted analysis of amino acids and organic acids

Fifty milligrams of LV tissue were frozen using liquid nitrogen, ground into a powder, and extracted with a solution of 70% methanol and hydroxylamine (1 mol·L^−1^) at pH 7.6. The following internal and external isotope‐labeled standards were added to the samples: ^13^C_3_‐lactic acid (400 nmol), ^13^C_3_‐pyruvic acid (20 nmol), ^13^C_4_‐β‐hydroxybutyric acid (20 nmol), ^13^C_4_‐α‐ketobutyric acid (1 nmol), D_4_‐citrate (20 nmol), ^13^C_4_‐α‐ketoglutaric acid (20 nmol), D_4_‐succinic acid (20 nmol), D_3_‐malic acid (40 nmol), ^13^C_4_‐acetoacetic acid (20 nmol), ^13^C_4_‐fumaric acid (20 nmol), ^13^C_3_‐alanine (150 nmol), ^13^C_2_‐glycine (50 nmol), ^13^C_5_‐valine (20 nmol), ^13^C_6_,^15^N‐leucine (10 nmol), ^13^C_6_‐isoleucine (10 nmol), ^13^C_5_,^15^N‐proline (5 nmol), ^13^C_5_‐methionine (15 nmol), ^13^C_5_,^15^N‐serine (50 nmol), ^13^C_4_,^15^N‐threonine (50 nmol), D_5_‐phenylalanine (15 nmol), ^13^C_4_,^15^N‐aspartic acid (100 nmol), ^13^C_5_,^15^N‐glutamic acid (400 nmol), ^13^C_6_‐arginine (150 nmol), ^13^C_9_‐tyrosine (10 nmol), ^13^C_6_‐histidine (20 nmol), ^13^C_5_,^15^N_2_‐glutamine (400 nmol), ^13^C_4_,^15^N_2_‐asparagine (15 nmol), ^13^C_11_,^15^N_2_‐tryptophan (5 nmol), ^13^C_3_,^15^N‐cysteine (40 nmol), and ^13^C_6_,^15^N_2_‐lysine (50 nmol). After agitation for 2 min in a sonication bath, the mixture was supplemented with zirconium oxide beads (2.8 mm diameter, Omni International, Kennesaw, GA) and homogenized using Bead Ruptor. Subsequently, the pH of the mixtures was adjusted to between 5 and 6 using hydrochloric acid (1 mol·L^−1^). The samples were incubated at 70 °C for 15 min and centrifuged at 22 000 ***g*** for 10 min. The supernatants were evaporated to near dryness. The samples were digested with 100% methanol (2 mL), dried with ammonium sulfate, filtered and rinsed with methanol, a combination of steps repeated twice. After centrifugation at 7000 ***g***, the supernatants were concentrated and the reduced volumes were transferred into GC–MS vials and dried. The dry samples were solubilized in pyridine, heated at 45 °C for 90 min, derivatization with *N*‐*tert*‐butyldimethylsilyl‐*n*‐methyltrifluoroacetamide at 90 °C for 4 h, and injected into an Agilent 6890 N chromatograph coupled with a 5975 N mass spectrometer operating in electronic ionization mode using helium as the carrier gas maintained at 7.7 mL·min^−1^ in split mode (5.6 : 1). The program temperature was set at 150 °C for 3 min, increased 7 °C·min^−1^ to 210 °C, held for 3 min, increased 7 °C·min^−1^ to 310 °C, held for 5.5 min, and finally increased 40 °C·min^−1^ to 320 °C. Metabolites were identified based on m/z ratios and retention times and quantified using internal and external standards and standard curves.

### Gene expression analyses

Total mRNA was extracted from the tissue using a two‐step protocol with the acid‐guanidium‐phenol‐based Trizol™ reagent (Invitrogen Canada Inc., Burlington, Ontario, Canada) followed by solid‐phase purification on columns with Aurum™ total RNA Mini Kit (Bio‐Rad, Mississauga, ON, Canada) to eliminate residual contaminants, such as genomic DNA. Reverse‐transcription to cDNA was performed in a total volume of 20 μL using deoxynucleoside triphosphate (dNTP) nucleotides and Moloney Murine Leukemia Virus (MMLV) Reverse Transcriptase (Invitrogen) at 37 °C for 75 min followed by 95 °C for 10 min. The sample volumes were diluted 1 : 10 in diethylpyrocarbonate (DEPC)‐treated water to remove RNases and kept at −20 °C until use. Real‐time quantitative polymerase chain reaction (RT‐qPCR) amplification was carried out in a 10 μL reaction mixture containing 1 μL of the cDNA template, 0.25 μL of each specific primer (20 μm stock), and 5 μL of SsofastTM EvaGreen® Supermix (Bio‐Rad Laboratories, Hercules, CA, USA) with the final volume adjusted with distilled water. The cycling protocol consisted of 40 cycles starting at 95 °C for 30 s, then at 60 °C for 90 s, and at 95 °C for 15 s using a QuantStudio 5 RT PCR system. Relative mRNA expression levels were determined using the comparative cycle threshold (*C*_t_) method ($2^{-\Delta \Delta C_{t}}$) using internal reference genes for normalization. Primer sequences are listed in Tables 1, 2. Additional primer sets (Table 2) were used to confirm the qPCR results. Data are expressed as fold changes relative to the corresponding 3 and 24‐h sham‐operated control mice.

**Table 1:** Murine primer sequences for qPCR.

Gene	Primer	Product length (bp)	NCBI gene ID
*Alox5*	Forward GTCCTGAGGGATGGACGTGCAAAAT Reverse TGCCGTGCCTCCAGTTCTTTACG	89	11689
*Alox12*	Forward AGTGCGTTTGTGGCTGGTTGGG Reverse AAGTCAAACTCCTCCTCCTTGCCCC	90	11684
*Alox15*	Forward TGGGGCAACTGGAAGGATGGCA Reverse AACGGTGTCCATTGTCCCCAGAAC	138	11687
*Cd36*	Forward TGGAGCAACTGGTGGATGGTTTCC	149	12491
	Reverse CTACGTGGCCCGGTTCTACTAATTCA		
*Cebpb*	Forward AAGAGCCGCGACAAGGCCAA	106	12608
	Reverse GCGACAGCTGCTCCACCTTCTT		
*Hprt*	Forward TCCTCCTCAGACCGCTTTTTGCC	80	15452
	Reverse CATCGCTAATCACGACGCTGGGA		
*Nfe2l2*	Forward ACCATGAGTCGCTTGCCCTGGAT	80	18024
	Reverse TGCCAAACTTGCTCCATGTCCTGC		
*Ppara*	Forward TTTGCTGTGGAGATCGGCCTGG	100	19013
	Reverse TGGTTGCTCTGCAGGTGGAGCTT		
*Ppard*	Forward ACACACGCTTCCTTCCAGCAGC	138	19015
	Reverse TTGTCCCCGCACACCCGACATT		
*Pparg*	Forward GCTGAACGTGAAGCCCATC	150	19016
	Reverse ACGTGCTCTGTGACGATCTG		
*Ppia*	Forward AGACTGAATGGCTGGATGGCAAGC	98	268373
	Reverse GCCATTCCTGGACCCAAAACGCT		

**Table 2:** Murine validation primer sequences for qPCR.

Gene	Primer	Product length (bp)	NCBI gene ID
*Alox5*	Forward CAGGGTCAAGAAGTTGGTGG Reverse CCGTGTAGGAGGGCATGACT	99	11689
*Alox12*	Forward GGAGAGGGAATCCTGAGCC Reverse GTGGCCCAGCAGTAGGT	125	11684
*Hprt*	Forward TTGGATACAGGCCAGACTTTGT	153	15452
	Reverse TGCGCTCATCTTAGGCTTTGT		
*Ppard*	Forward ATGAAGACAAACCCACGGTAAAG	118	19015
	Reverse CTGTGGCTGTTCCATGACTGAC		

### Statistical analyses

Analyses were conducted using graphpad prism (version 10.4.2; San Diego, CA, USA). Data are presented as mean ± SEM. Normality and homogeneity of variances were evaluated using Statistics Kingdom (www.statskingdom.com). When required to meet the assumptions of the statistical tests, analyses were conducted on log‐transformed data. Group comparisons were conducted using either Student's *t*‐test or two‐way ANOVA with time and treatment as factors, followed by Fisher's least significant difference (LSD) *post hoc* test for multiple comparisons. When the treatment × time interaction was significant, simple effects were assessed at each time point. Potential outliers were assessed using Grubbs' test. Data with a *P*‐value <0.05 (*α* = 0.05) were excluded from subsequent analyses as indicated in the figures. Statistical significance was defined as *P* < 0.05.

## Results and discussion

### Azapeptide MPE‐298 reduces left ventricular injury after myocardial ischemia and reperfusion

The cardioprotective effect of azapeptide MPE‐298 was initially investigated by administration of a single IV dose shortly before reperfusion. Hypothesizing that azapeptide MPE‐298 would act in a CD36‐dependent manner, both *Cd36* ^*+/+*^ and *Cd36* ^−/−^ mice were subjected to LCAL and reperfusion for 24 h to assess myocardial lesions. Ten minutes before reperfusion, mice received an IV injection of either vehicle (0.9% NaCl) or azapeptide MPE‐298 (3 μmol·kg^−1^) (Fig. 1A). The selected dose was based on prior dose–response experiments, which demonstrated a dose‐dependent reduction in infarct size [30]. The AAR relative to the LV, quantified using the area‐weighted method, was consistent across genotypes and treatment groups, averaging 47%. This uniform AAR confirms that all animals experienced a comparable ischemic insult and demonstrates strong experimental reproducibility (Fig. 1B,C). An AAR of approximately 50% of the LV indicates that the ligature was placed proximally, near the origin of the LAD, thereby producing a large ischemic territory. In *Cd36* ^+^^/^^+^ mice, treatment with MPE‐298 significantly reduced the IA relative to LV by 42%, from 16.2 ± 1.4% in vehicle‐treated mice to 9.4 ± 0.6% in MPE‐298‐treated mice (*P* < 0.01; Fig. 1B). When expressed as IA relative to AAR, MPE‐298 treatment produced a 44% reduction, from 35.9 ± 3.3% to 20.0 ± 1.5% (*P* < 0.001; Fig. 1B). These findings are consistent with the expected pattern in which the necrotic infarct develops within the AAR, which contains both viable and necrotic myocardium. In contrast, no significant IA reduction was observed in *Cd36* ^−/−^ mice (Fig. 1C), indicating that the cardioprotective effect of MPE‐298 is CD36‐dependent. The protective effect was further demonstrated in representative TTC‐stained mid‐ventricular sections, where MPE‐298‐treated *Cd36* ^+/+^ mice exhibited visibly smaller infarct area (white regions) compared with vehicle‐treated mice or with *Cd36* ^−/−^ mice irrespective of treatment (Fig. 1D). These images are qualitative and are provided to complement the quantitative infarct‐area analysis.

**Fig. 1:**
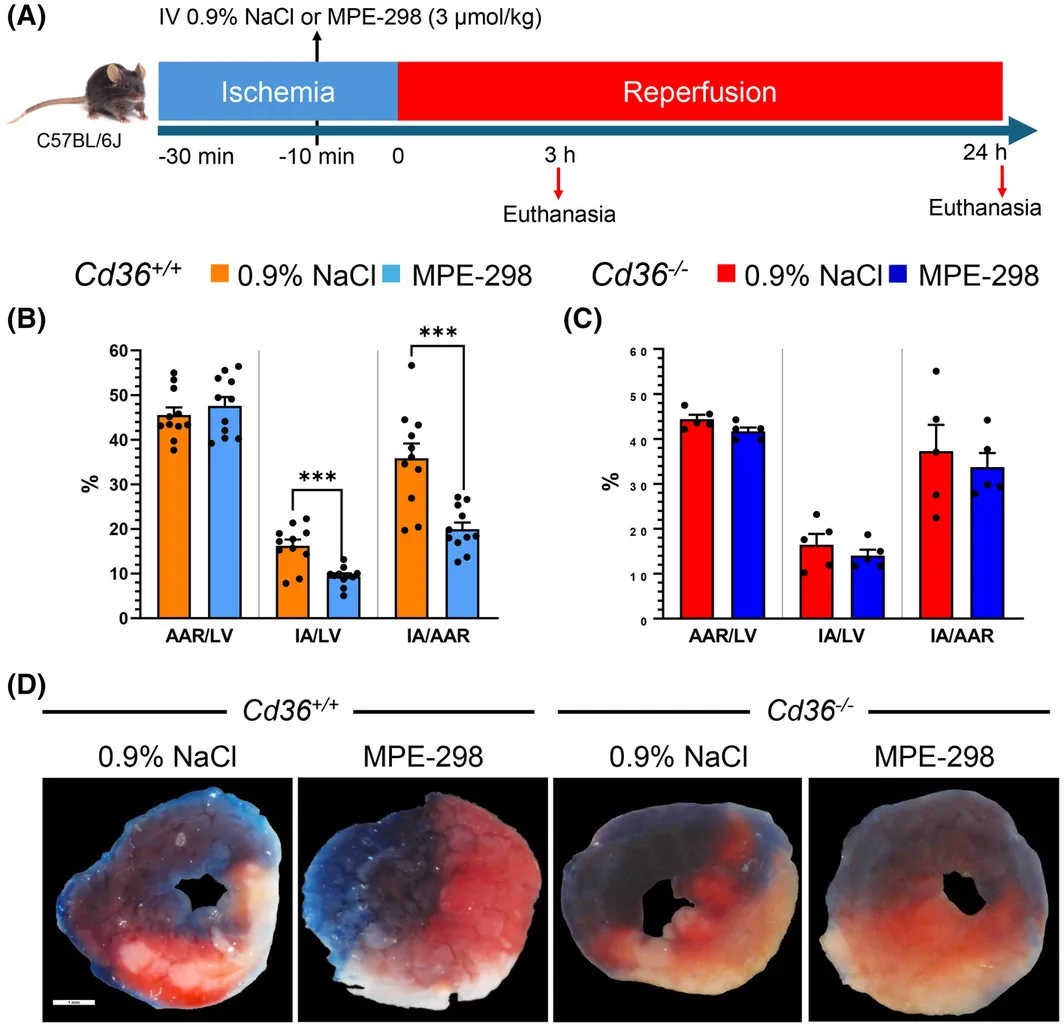
Treatment with MPE‐298 reduces infarct area in *Cd36* ^+/+^ mice but not in *Cd36* ^−/−^ mice after myocardial ischemia and reperfusion (MI/R) (A) Experimental timeline: mice underwent LCAL for 30 min, followed by reperfusion and monitoring at 3 and 24 h. Azapeptide MPE‐298 (3 μmol·kg^−1^) or vehicle (0.9% NaCl) were administered IV 10 min before reperfusion. (B, C) Quantification of myocardial infarct area at 24 h post‐MI/R. Bar graphs and dot plots show AAR/LV, expressed as a percentage of LV area (left panel); IA/LV expressed as a percentage of LV area (middle panel), and IA/AAR as a percentage of AAR (right panel) in *Cd36* ^+/+^ mice (*n* = 11/group) and *Cd36* ^−/−^ mice (*n* = 5/group). (D) Representative TTC‐ and Evans blue‐stained mid‐ventricular cross‐sections from *Cd36* ^+/+^ and *Cd36* ^−/−^ mice showing the AAR (red and white), infarcted tissue (white), and nonischemic tissue (blue) 24 h after reperfusion. Data are mean ± SEM. ***P < 0.001, unpaired *t*‐test. Scale bar, 1 mm. Area at risk, AAR; infarct area, IA; LCAL, left coronary artery ligation; LV, left ventricle.

Administration of a single dose of azapeptide MPE‐298 shortly before reperfusion provided acute protection against LV injury in a CD36‐dependent manner. In earlier studies, a 14‐day pretreatment with either a non‐selective linear CD36 ligand EP 80317, or a selective linear azapeptide CP‐3(iv), administered daily at 0.3 to 1 μmol·kg^−1^ prior to a 30‐min ischemic period, were found to reduce the IA to LV area ratio by 34% and 56%, respectively [11, 30]. Such attenuations of LV injury were largely attributed to a decreased myocardial lipid burden, linked to transiently lower plasma NEFA levels resulting from reduced lipolysis [11]. The CP‐3(iv) pretreated mice were also shown to exhibit increased expression of adiponectin in adipose tissue and higher circulating adiponectin levels [30]. In addition, administering CP‐3(iv) 10 min before reperfusion induced a dose‐dependent reduction of the IA to LV area by 44% at the highest dose tested (1 μmol·kg^−1^) compared with vehicle‐treated CD36^+/+^ mice.

### Azapeptide MPE‐298 attenuates LCFA accumulation in LV after myocardial ischemia and reperfusion

Cellular uptake of LCFA is primarily governed by CD36 at the cell membrane (sarcolemma) [31]. The effects of azapeptide MPE‐298 on FA composition and abundance were consequently examined in the LV after reperfusion. Following MI, reperfusion of salvageable ischemic myocardium leads to a rapid restoration of FA uptake and oxidation [10]. This early rise in LCFA β‐oxidation disrupts the balance between glucose and FA oxidation as competing sources of adenosine triphosphate (ATP) production [32]. The predominance of β‐oxidation requires more oxygen for ATP production and leads to excess hydrogen ions production, which in turn contributes to ionic imbalances that decrease heart efficiency [33]. Transient inhibition of early uptake of LCFA after reperfusion may protect the heart from detrimental oxidative stress after reperfusion and improve contractile recovery. Consistent with this hypothesis, mice pretreated with the GHRP peptide EP 80317 were shown to exhibit reduced myocardial FA uptake 6 h after reperfusion by micro‐positron emission tomography (μPET) using [^18^F]‐labeled fluoro‐6‐thia‐heptadecanoic acid as a tracer of sarcolemmal and mitochondrial NEFA uptake [11].

Targeted analysis of FA was performed after esterification as methyl esters (FAMES). Analysis of fold changes in the LV revealed that, at 3 h postreperfusion, mice receiving a single IV dose of the azapeptide MPE‐298 showed transient reductions in certain LCFA compared with vehicle‐treated controls. The decrease was observed across all classes of saturation of FA: saturated (SFA), monounsaturated (MUFA), and polyunsaturated (PUFA, heatmap Fig. 2A). Significant reductions were observed compared with those of vehicle‐treated mice in the relative abundance of SFA [myristic, pentadecanoic and palmitic acids (Fig. 2B–D)], MUFA [palmitoleic, vaccenic and oleic acids (Fig. 2E–G)], and PUFA [arachidonic, eicosatrienoic (ETE) and docosahexaenoic (DHA) acids (Fig. 2H–J)]. Modest downward trends were observed for docosapentaenoic (DPA) and linoleic acids (Fig. 2K,L). SFA have long been considered detrimental to cardiovascular health, and dietary recommendations to limit their intake have persisted. Although elevated total SFA concentrations have been linked to higher cardiovascular risk, further studies showed that individual SFA subtypes did not behave consistently, with some positively associated with greater risk while others showed inverse association [34]. Whereas the long‐term health effects of SFA and some MUFA remain actively debated, the benefits of PUFA are generally more widely supported [35], though not without some contradictory findings [36]. Importantly, the changes in LCFA levels within the LV after treatment with azapeptide MPE‐298 were transient. By 24 h, the values returned to control (vehicle) levels. At this time point, levels of vaccenic acid and DPA were higher than those in vehicle‐treated mice. Although speculative, this pattern may reflect an adaptive response favoring the retention of specific MUFA and PUFA.

**Fig. 2:**
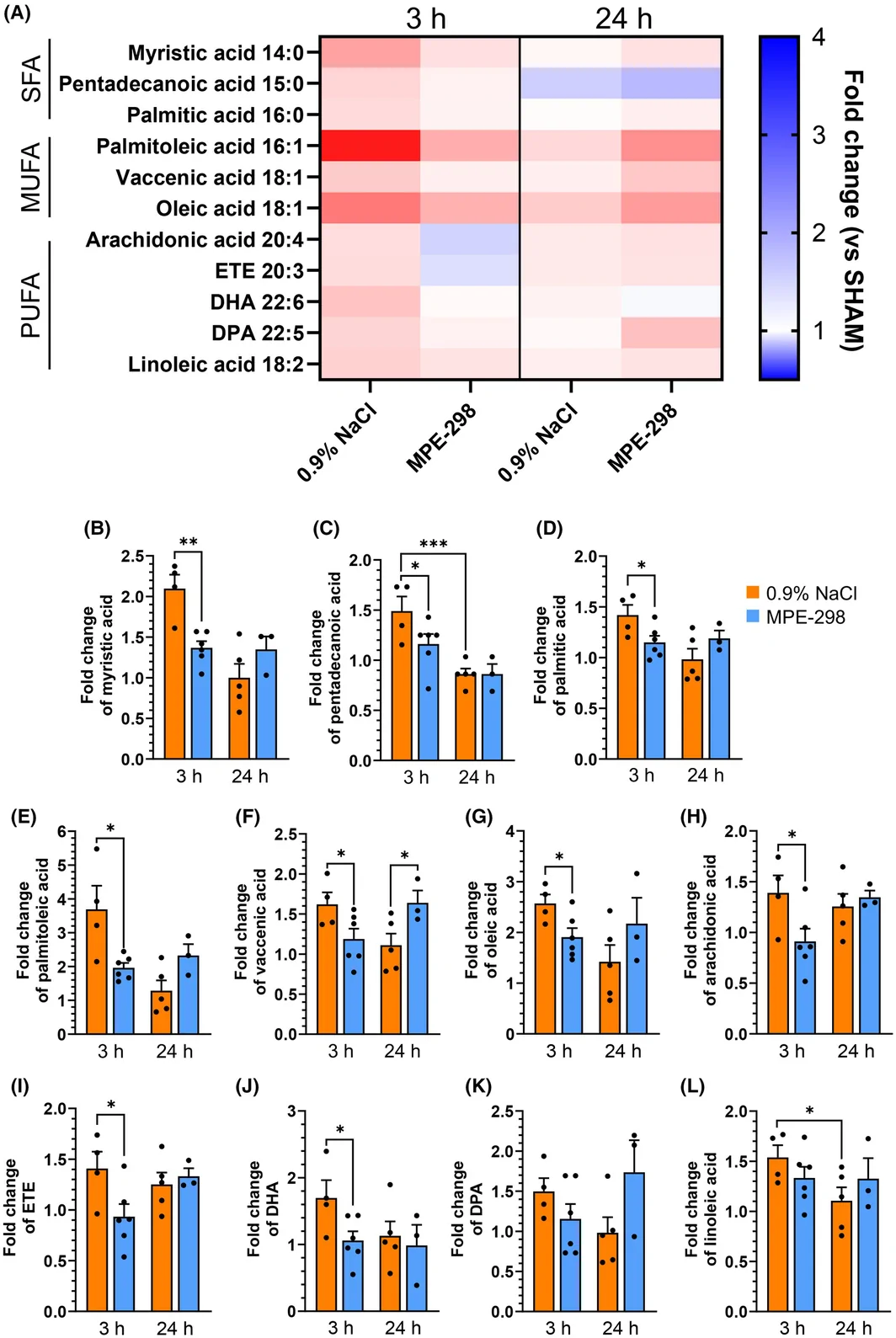
Administration of a single IV dose of azapeptide MPE‐298 (3 μmol·kg^−1^) prior to reperfusion induces a transient reduction of the relative LV content of LCFA at 3 h postreperfusion in mice that underwent a temporary left coronary artery ligation (LCAL) for 30 min. (A) Heatmap depicting the relative LCFA content in the LV of vehicle‐ and MPE‐298‐treated mice at 3 and 24 h postreperfusion. Bar graphs (B–L) represent LV content of LCFA normalized to the values in sham‐operated mice, including (B) myristic acid, (C) pentadecanoic acid, (D) palmitic acid, (E) palmitoleic acid, (F) vaccenic acid, (G) oleic acid, (H) arachidonic acid, (I) eicosatrienoic acid (ETE), (J) docosahexaenoic acid (DHA), (K) docosapentaenoic acid (DPA) and (L) linoleic acid. Data are expressed as the mean ± SEM of 4–6 mice. Statistical analysis was performed using a two‐way ANOVA with treatment and time as independent variables, followed by Fisher's LSD post hoc test. *P < 0.05, **P < 0.01, ***P < 0.001. LCFA, long chain fatty acids; LV, left ventricle; MUFA, monounsaturated fatty acids; PUFA, polyunsaturated fatty acids; SFA, saturated fatty acids.

Notably, levels of SFA, MUFA, and linoleic acid declined between 3 and 24 h in vehicle‐treated mice, suggesting that the peak in LCFA uptake occurs primarily during the early reperfusion phase, consistent with previous reports [37]. In sum, azapeptide MPE‐298 reduced transiently myocardial LCFA content independent of saturation state in the LV of mice subject to MI/R. Previous findings in mice pretreated with the CD36 ligand EP80317 showed transient suppression of peripheral lipolysis reducing circulating NEFA levels at 6 h following MI/R [14]. In the present study, plasma NEFA levels showed a modest but nonsignificant decline at 3 h, while plasma cardiac troponin remained unchanged (1.70 ± 0.44 mmol·L^−1^ in vehicle‐treated mice and 1.52 ± 0.33 mol·L^−1^, *n* = 3 per group). Consistent with our earlier findings, reduced lipolysis was linked to lower cardiac FA burden, and this correlation prompted us to assess markers of lipolysis in epididymal fat.

### Azapeptide MPE‐298 alters expression of lipolysis‐related genes in epidydimal adipose tissue postmyocardial ischemia and reperfusion

Lipolysis is acutely activated in response to MI/R‐induced oxidative stress and inflammation [38]. The transcription factor nuclear factor erythroid‐derived 2‐like 2 (Nfe2l2), also known as Nrf2 (but distinct from nuclear respiratory factor 2), is a key regulator of cellular antioxidant defense mechanisms which are activated in response to cellular stress, such as those during MI/R injury [39]. When blood flow is restored, the sudden reactivation of the mitochondrial electron transport chain becomes a major source of ROS, and additional contributors including xanthine oxidase and NADPH oxidase (NOX) further amplify reperfusion‐driven oxidative stress. Early activation of Nfe2l2 has, however, been shown to worsen MI/R injury by amplifying cardiac inflammation, enhancing the release of pro‐inflammatory cytokines and driving macrophages toward a pro‐inflammatory M1 phenotype [40]. Consistent with this, mice administered a single IV dose of azapeptide MPE‐298 (3 μmol·kg^−1^) showed a transient reduction in *Nfe2l2* gene expression in epididymal adipose tissue at 3 h postreperfusion (Fig. 3A) accompanied by decreased expression of downstream target genes, including CCAAT/enhancer‐binding protein beta (Cebpβ) [41] and CD36 [42] (Fig. 3B,C). The transcription factor C/EBPβ has also been shown to transcriptionally upregulate CD36 mRNA expression [43]. Knockdown of CD36 in 3T3‐L1 adipocytes has been linked to decreased lipolytic activity and reduced phosphorylation of key lipolytic enzymes, including hormone sensitive lipase and perilipin [44]. The functional importance of CD36 expression in adipose tissue is further supported by the reduction of lipolysis following treatment with the CD36 inhibitor sulfo‐*n*‐succinimidyl oleate [44]. Notably, at 24 h, *Nfe2l2* expression in vehicle‐treated mice showed a nonsignificant decrease relative to the 3‐h time point, whereas its downstream target genes were reduced in the vehicle‐treated groups but remained unchanged following treatment (Fig. 3A–C). A 14‐day pretreatment protocol with the linear azapeptide CP‐3(iv) (0.3 μmol·kg^−1^) elicited a transient increase in *Nfe2l2*, *Cebpβ*, and *CD36* expression in epididymal fat at 6 h postreperfusion [30]. Unlike the single‐dose administration used in the present study, this prolonged exposure may account for the divergent transcriptional responses. However, aside from treatment duration, differences in treatment and treatment regimen, sampling time, and administered dose may also account for these observations.

**Fig. 3:**
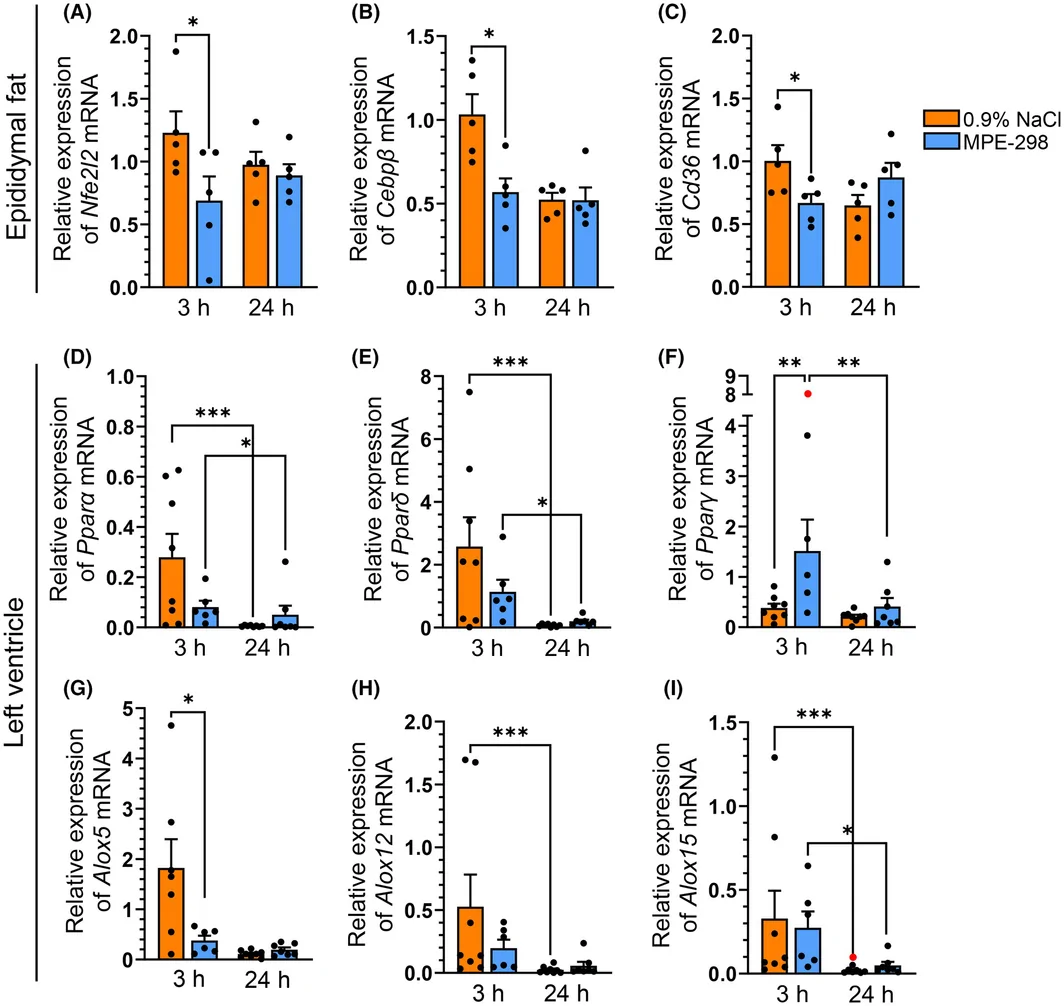
Administration of azapeptide MPE‐298 reduces the expression of genes involved in metabolic and inflammatory pathways post‐MI/R. Mice were treated IV 10 min prior to reperfusion with either 0.9% NaCl or MPE‐298 (3 μmol·kg^−1^) and monitored at 3 or 24 h postreperfusion. Bar graphs show the relative mRNA levels in adipose tissue: (A) *Nfe2l2*, (B) *Cebpβ* and (C) *Cd36*, and in LV: (D) *Pparα*, (E) *Pparδ*, (F) *Pparγ*, (G) *Alox5*, (H) *Alox12*, and (I) *Alox15*. Data are expressed as the mean ± SEM of 5–8 mice. Statistical analysis was performed using a two‐way ANOVA with treatment and time as independent variables, followed by Fisher's post hoc test. Outliers (red dots) were identified using the Grubbs' test. *P < 0.05, **P < 0.01, ***P < 0.001. MI/R, myocardial ischemia and reperfusion.

The LCFA serve as endogenous ligands of peroxisome proliferator‐activated receptor (PPAR) subclasses, with PPARα being the most abundantly expressed isoform in the heart [45]. Playing a key role in regulating mitochondrial lipid β‐oxidation, PPARα promotes fatty acid oxidation (FAO) and concurrently reduces glucose utilization in cardiomyocytes [45]. Our results align with the sequence whereby MI/R triggers early inflammation and myocardial ROS, leading to increased PPARα expression in the myocardium. Temporarily reducing PPARα, and potentially PPARδ, expression (Fig. 3D,E) during the early hours of reperfusion may be advantageous, because premature enhancement of β‐oxidation may compromise cardiac efficiency. At later time points, however, PPARα signaling supports metabolic recovery. In contrast, PPARγ agonists have been reported to exert cardioprotective effects in models of MI/R, supporting a role for PPARγ as a key transcription factor mediating antioxidative and anti‐inflammatory responses, while improving myocardial energy metabolism [46, 47]. Treatment with MPE‐298 showed an increase in PPARγ expression (Fig. 3F), indicating a potential beneficial effect toward inflammation resolution and cardioprotection.

Animal models have controversially shown that myocardial MI/R results in elevated production of leukotrienes in the myocardium [48]. Growing evidence indicates that metabolites derived from the 5‐lipoxygenase (5‐LO) pathway contribute negatively to myocardial functional recovery. Previously, pretreatment with EP 80317 was found to selectively downregulate *Alox5* gene expression and to reduce leukotriene B_4_ (LTB_4_) levels in the lungs of mice subjected to transient skeletal ischemia–reperfusion, suggesting potential for modulating the inflammatory response [49]. Noting the transient reduction of levels of the LCFA arachidonic acid in the LV at 3 h after reperfusion (Fig. 2H), we investigated the effect of azapeptide MPE‐298 on the *Alox5* mRNA levels following MI/R. Azapeptide administration led to a transient reduction in *Alox5* mRNA expression in mice LV (Fig. 3G), without significantly changing mRNA levels of *Alox12* (Fig. 3H) and *Alox15* (Fig. 3I). In the early response to MI/R, metabolites from Alox5 may play roles which are transiently suppressed upon azapeptide treatment. Previously, LTB_4_, a lipid mediator of inflammation, was shown to be an endogenous ligand for PPARα [50]. Reduction in *Alox5* expression at 3 h after reperfusion may decrease transiently activation of PPARα. The mechanistic links between ALOX5, CD36, and PPARα warrant further investigation in the context of MI/R injury.

### Azapeptide MPE‐298 alters amino acid composition in the left ventricle following myocardial ischemia and reperfusion

In the early phase of reperfusion following ischemia, we observed that the LV of mice treated with MPE‐298 exhibited distinctly reduced levels of LCFA (Fig. 2). Previously, energy metabolism in the heart after reperfusion was shown to shift toward a greater reliance on glucose [37] and potentially anaplerosis of specific amino acids [51]. In addition to serving as metabolic substrates in MI/R injury, certain amino acids may play broader roles, such as modulating the cellular responses to stress. The influence of azapeptide MPE‐298 on levels of amino acids and tricarboxylic acid (TCA) cycle intermediates was examined in the LV of mice subjected to MI/R (heatmaps Fig. 4A,B). Among TCA metabolites, plasma levels of 2‐hydroxyglutarate, fumarate and malate have been prospectively associated with increased cardiovascular risk [52]; notably the relative levels of these intermediates in the LV tended to decrease in MPE‐298‐treated mice (Fig. 4B). The amino acid glutamate serves as a substrate that fuels the TCA cycle through conversion to α‐ketoglutarate via transamination (Fig. 4C) [53]. Glutamine (Fig. 4D) and proline (Fig. 4E), which can be metabolized to glutamate [54], have been similarly shown to contribute to the production of α‐ketoglutarate [55]. Glutamate, cysteine and glycine are components in the synthesis of glutathione, a potent intracellular antioxidant [56]. In the LV of mice treated with azapeptide MPE‐298 at 3 h after reperfusion, glutamate and cysteine declined transiently, and glutamine, proline and glycine all showed modest trends toward reduction (Fig. 4E–G). Moreover, the aromatic amino acids, tyrosine (Fig. 4H) and phenylalanine (Fig. 4I), were reduced at 3 and 24 h in MPE‐298‐treated compared with vehicle‐treated mice. Although metabolism of tyrosine and phenylalanine by way of aromatic ring hydroxylation may lead to the TCA intermediate fumarate [57], levels of the latter were not significantly altered (Fig. 4B). Amino acids serve many roles beyond energy metabolism, including protein synthesis, cofactors in biochemical reactions and signaling intermediates [58]. For example, a trend toward reduced LV alanine levels was observed (Fig. 4J). Alanine is transaminated to pyruvate [51], yet neither pyruvate (Fig. 4K) nor lactate (Fig. 4L) differed significantly between MPE‐298‐treated and vehicle‐treated mice suggesting minimal disruption of this metabolic pathway early after reperfusion. Further studies are required to determine how the modest alterations contribute to the response to MPE‐298 at the different time points.

**Fig. 4:**
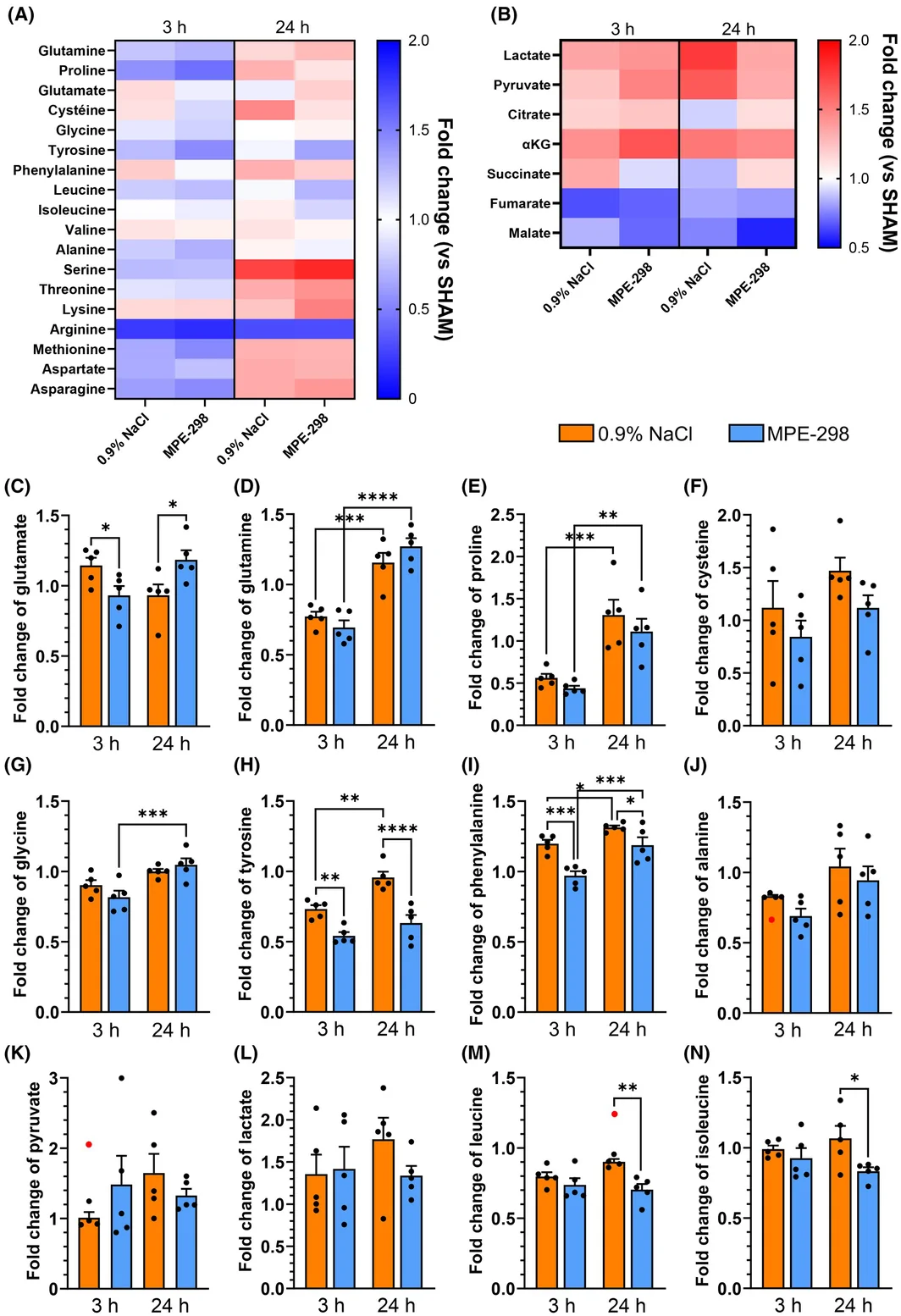
Single IV dose of azapeptide MPE‐298 (3 μmol·kg^−1^) administered before reperfusion induced a transient reduction in the left ventricle (LV) content of specific amino acids at 3 and 24 h postreperfusion in mice that underwent a temporary left coronary artery ligation (LCAL) for 30 min. Heatmaps depicting fold change relative to corresponding sham‐operated mice of (A) amino acids and (B) tricarboxylic acid (TCA) intermediates in the LV of vehicle‐ and MPE‐298‐treated mice at 3 and 24 h postreperfusion. Bar graphs represent the relative LV content of specific amino acids and metabolites: (C) glutamate, (D) glutamine, (E) proline, (F) cysteine (G) glycine (H) tyrosine, (I) phenylalanine, (J) alanine, (K) pyruvate, (L) lactate, (M) leucine and (N) isoleucine. Data are expressed as the mean ± SEM of 4–5 mice. Statistical analysis was performed using a two‐way ANOVA with treatment and time as independent variables, followed by Fisher's post hoc test. Outliers (red dots) were identified using the Grubbs' test. *P < 0.05, **P < 0.01, ***P < 0.001. αKG, alpha‐ketoglutarate.

The branched‐chain amino acids (BCAA) leucine, isoleucine, and valine are essential and must be obtained through diet. They contribute minimally to normal cardiac energy production, but MI/R impairs their catabolism leading to BCAA accumulation, disturbed cardiac metabolic homeostasis and stress [59, 60]. Although no significant changes in LV content of BCAA were observed between vehicle and MPE‐298 groups at 3 h after reperfusion (Fig. 4M,N), by 24 h, both leucine and isoleucine levels were reduced, indicating little myocardial accumulation but suggesting potential roles in myocardial metabolism during the critical early reperfusion period.

The limitations of this study are due in part to examination of only two time points after reperfusion and only male animals. Despite these constraints, cardioprotective effects were evident after administration of a single dose of azapeptide MPE‐298 prior to reperfusion. The study revealed the early beneficial impact of azapeptide treatment on myocardial metabolism specifically the reduction of LCFA following MI/R. The beneficial effects on myocardial metabolism complement earlier findings from studies featuring pretreatment with a CD36 ligand which, respectively, reduced and increased circulating NEFA and adiponectin levels with increased expression of the latter in adipose tissue [11, 30]. Selective CD36 targeting has shown additional therapeutic benefit for treating MI/R injury [30].

In conclusion, our findings suggest a potential mechanism by which MPE‐298 mediates cardioprotection after MI/R by modulating cardiac substrate metabolism and inflammatory signaling. Specifically, targeted FAMES profiling revealed a transient and early reduction in LCFA accumulation in the LV, independent of saturation state. This metabolic shift was accompanied by a temporary pronounced downward trend in *PPARα/PPARδ* expression, known to promote FAO. In contrast, *PPARγ* expression was temporarily increased in the LV, consistent with its established role in suppressing pro‐inflammatory pathways and improving myocardial energy metabolism. Moreover, we observed a reduction in *Alox5* expression, further supporting an anti‐inflammatory effect of MPE‐298. For the first time, our study also characterized amino acid changes during reperfusion in mice treated with MPE‐298, revealing modest but noteworthy alterations, including amino acids involved in energy production and antioxidant defense. Reduced relative BCAA levels in the LV at 24 h, which may represent a secondary metabolic effect worth exploring in future studies. Collectively, these results indicate that MPE‐298 may protect the myocardium by transiently modulating metabolic and inflammatory pathways during early reperfusion.

## Conflict of interest

The authors declare no conflict of interest.

## Author contributions

SM conceived and designed the experiments. S‐PG, SM, and HO supervised the studies. WDL and AA provided resources. JG, NR, LM, MV, and DNH performed the experiments and analyzed the data. CD and MR performed the GC–MS and provided the results. S‐PG, SM, and WDL wrote the manuscript. WDL edited the manuscript. ACC, HO, DNH, LM, NR, and JG made manuscript revisions. S.M. and S.‐P.G. are co‐senior authors.

## Data Availability

All data underlying the results reported in this manuscript can be obtained by contacting the corresponding author upon reasonable request.
